# Coagulation parameters predict COVID-19-related thrombosis in a neural network with a positive predictive value of 98%

**DOI:** 10.3389/fimmu.2022.977443

**Published:** 2022-09-28

**Authors:** Romy de Laat-Kremers, Raf De Jongh, Marisa Ninivaggi, Aernoud Fiolet, Rob Fijnheer, Jasper Remijn, Bas de Laat

**Affiliations:** ^1^ Department of Data Analysis and Artificial Intelligence, Synapse Research Institute, Maastricht, Netherlands; ^2^ Department of Anesthesiology, Ziekenhuis Oost Limburg, Genk, Belgium; ^3^ Department of Anesthesiology, Fondation Hopale, Berck-sur-Mer, France; ^4^ Department of Functional Coagulation, Synapse Research Institute, Maastricht, Netherlands; ^5^ Department of Internal Medicine, Meander Medical Center, Amersfoort, Netherlands; ^6^ Department of Clinical Chemistry, Meander Medical Center, Amersfoort, Netherlands

**Keywords:** thrombin generation, neural network, COVID-19, thrombosis, prediction

## Abstract

Thrombosis is a major clinical complication of COVID-19 infection. COVID-19 patients show changes in coagulation factors that indicate an important role for the coagulation system in the pathogenesis of COVID-19. However, the multifactorial nature of thrombosis complicates the prediction of thrombotic events based on a single hemostatic variable. We developed and validated a neural net for the prediction of COVID-19-related thrombosis. The neural net was developed based on the hemostatic and general (laboratory) variables of 149 confirmed COVID-19 patients from two cohorts: at the time of hospital admission (cohort 1 including 133 patients) and at ICU admission (cohort 2 including 16 patients). Twenty-six patients suffered from thrombosis during their hospital stay: 19 patients in cohort 1 and 7 patients in cohort 2. The neural net predicts COVID-19 related thrombosis based on C-reactive protein (relative importance 14%), sex (10%), thrombin generation (TG) time-to-tail (10%), α_2_-Macroglobulin (9%), TG curve width (9%), thrombin-α_2_-Macroglobulin complexes (9%), plasmin generation lag time (8%), serum IgM (8%), TG lag time (7%), TG time-to-peak (7%), thrombin-antithrombin complexes (5%), and age (5%). This neural net can predict COVID-19-thrombosis at the time of hospital admission with a positive predictive value of 98%-100%.

## Introduction

In December 2019, Severe Acute Respiratory Syndrome-CoV-2 (SARS-CoV-2) virus emerged and caused a pandemic that led to hospitalization of over one hundred thousand patients worldwide (1). By November 2021, more than 250 million SARS-CoV-2 infections have been reported and the disease caused over 5.1 million deaths (2). The course of the disease differs greatly among patients: some patients have symptoms resembling a mild cold or are even asymptomatic, while others suffer from fulminant pneumonia requiring hospitalization and admission to the intensive care unit (ICU) (3, 4).

An important clinical complication of COVID-19 infection is thrombosis, indicating a role for the coagulation system in the pathogenesis of COVID-19 (5, 6). It was previously reported that severe COVID-19 patients have decreased antithrombin levels and increased levels of fibrinogen, fibrin degradation products and D-dimer (1). The degree of elevation correlates with the severity of the disease. Currently, COVID-19 patients admitted to the ICU are treated with low molecular heparin (LMWH) to prevent the development of thrombi that could lead to thrombo-embolism or stroke.

There have been several attempts to predict and thereby prevent thrombosis in COVID-19 patients. Due to multifactorial nature of thrombosis, one lab result is not sufficient to predict thrombosis (7). Therefore, it is unlikely that the occurrence of thrombosis in COVID-19 patients can be accurately predicted by a single lab test parameter or a combination of two. The neural net is an artificial intelligence tool that focusses on the integration of data to predict a certain outcome, in our case the occurrence of thrombosis during a COVID-19 infection (7). Since the start of the SARS-CoV-2 pandemic, many neural nets have been developed by researchers worldwide (8–17). Most of these neural nets focus on the diagnosis of COVID-19, and the neural net’s input is either an X-ray (9–13) or a computed tomography (CT)-scan of the chest (8) to study the effect of COVID-19 in the lungs. Other neural networks make predictions on a national or international level, by forecasting the COVID-19 spread through a geographical region (14–17). Additionally, researchers from the Wuhan region have shown that a neural net based on C-reactive protein, lactate dehydrogenase and lymphocyte count can predict mortality in Chinese COVID-19 patients (18).

Our goal was to use the neural networking approach to predict the risk of thrombosis during COVID-19 infection based on lab test results obtained at the time of hospital admission. Therefore, we collected blood samples of COVID-19 patients at 2 different hospitals, one population enrolled patients at the time of hospital admission (cohort 1) and one population enrolled patients admitted to the ICU (cohort 2) (19, 20). As input for our thrombosis-prediction neural network we focused on functional coagulation tests, such as the thrombin and plasmin generation, coagulation factor levels and markers of inflammation.

## Materials and methods

### Patients

COVID-19 patients were included at two centers (Meander Medical center in Amersfoort and Hospital Oost-Limburg in Genk) after approval of the “MEC-U” and the “Comité Medische Ethiek” medical ethics committees, respectively, and in accordance with the declaration of Helsinki. In the first cohort, COVID-19 patients were enrolled in the study at the time of hospital admission, and selected for analysis after COVID-19 infection was confirmed by a positive PCR test (n=133) (19). In the second cohort, intensive care unit (ICU) patients were included if they when tested positive for COVID-19 (n=16) (20). Samples were taken after informed consent of the patient or its legal relative. Blood was taken by venipuncture (cohort 1) or arterial catheter (cohort 2). Plasma was prepared by centrifuging twice for ten minutes at 2630g and stored at - 80°C until further analysis. In both cohorts, thrombosis was defined as pulmonary embolism, deep vein thrombosis as diagnosed by ultrasound, acute coronary syndrome, a cerebral ischemic attack or mesenteric ischemia.

### Thrombin generation

TG was measured by Calibrated Automated Thrombinography (CAT) using PPP reagent, calibrator and FluCa from Diagnostica Stago (France). TG was measured in the presence or absence of thrombomodulin (TM; the concentration causing 50% inhibition of the peak height in pooled normal plasma; Synapse Research Institute, the Netherlands) to test the sensitivity of the activated protein C (APC) system. In the cohort of ICU patients, who were all treated with low molecular weight heparin, heparin was neutralized with 0.045 mg/mL polybrene added to the plasma prior to the measurement of TG. In addition to the classical TG parameters ETP, peak height, time-to-peak and lag time, novel time-to-tail and curve width were quantified. The time-to-tail was quantified as the time it takes until the thrombin concentration stays below 1 nM at the end of thrombin generation. The curve width was quantified as the difference between the lag time and time-to-tail, providing a measure for the broadness of the curve.

### Thrombin dynamics

Prothrombin conversion curves were quantified form TG curves using the thrombin dynamics method (21). The following parameters were quantified from the prothrombin conversion curves: PC_tot_ (total amount of prothrombin converted during the TG test), PC_max_ (maximum rate of prothrombin conversion), T-AT (amount of thrombin-antithrombin complexes formed) and T-α_2_M (amount of thrombin-α_2_M complexes formed).

### Coagulation factor determinations

Plasma antithrombin, fibrinogen, FVIII, protein C and D-dimer levels were measured on the STA-R Max using the STA Stachrom AT III, STA Fibrinogen, STA Deficient FVIII, STA Staclot Protein C, and STA Liatest DDI kits, respectively, and in accordance with the manufacturers recommendations (Diagnostica Stago, France). Functional α_2_M levels were measured as previously described (Synapse Research Institute, the Netherlands) (21). VWF, active VWF, VWF pro-peptide were measured in house by ELISA as previously reported (22). C-reactive protein (CRP) levels were determined on the Architect C16000 according to the manufacturers specifications (Abbott, USA). Activated partial thromboplastin time (APTT) and cell counts were measured on the Sysmex CS-5100 using Innovin and Actin FSL reagents and Sysmex XN-9000 (Siemens, Germany) respectively. COVID-19 IgM titers were determined on the Afias-6 according to the manufacturers recommendations (Boditech, Republic of Korea).

### Plasmin generation assay

Plasmin generation was performed as previously described (23). Plasmin generation lag time, i.e. the time it takes until plasmin in formed, was quantified to serve as an input for the neural network.

### Neural network development

Matlab was used to develop and train neural networks using the neural pattern recognition application of the neural network toolbox (Mathworks, the Netherlands). The input of the “thrombosis or no thrombosis” classification network consisted of CRP, IgM titer for COVID-19, α_2_-Macroglobulin, thrombin-α_2_-Macroglobulin complexes, thrombin generation parameters (lag time, time-to-peak, time-to-tail and curve width) and plasmin lag time (**Table 2**). Parameters were selected based on (statistically insignificant) trends that were visible in cohort 1 and 2 and the crude (continuous) values for each parameter were used.

The hospital admissions cohort (cohort 1) was used to train the neural network, perform the initial validation and test the performance. The thrombosis group was much smaller than the non-thrombosis group (114 vs 19 subjects) in cohort 1. Subsequently, the thrombosis group was oversampled by 6-fold to overcome the difference in group size. The analysis was run 10 times and the model performance was quantified as the mean ± standard deviation of the 10 replicate analysis. The positive and negative predictive values (PPV and NPV) were calculated for each neural network to determine its diagnostic accuracy. Finally, the validity of the neural network was tested in the ICU cohort (cohort 2), which was acquired separately at another hospital to ensure the generalizability of the neural network to other patient cohorts.

### Statistical analysis

Statistical analyses were performed in GraphPad Prism (version 8, San Diego, USA). The Mann Whitney test was used to compare differences between groups and statistical significance was reported as p-values below 0.05.

## Results

In a total of 149 confirmed COVID-19 patients available from two study cohorts (19, 20), we quantified general laboratory parameters, the function of the coagulation system, coagulation factor levels and biomarkers of inflammation (**Tables 1**, **2**). We studied two cohorts of COVID-19 patients (**Tables 1**, **2**). Cohort 1 consisted of 133 COVID-19 patients enrolled in the study when they were admitted to the hospital for suspicion of COVID-19 infection and were tested positive for COVID-19 by PCR testing (**Table 1**). Cohort 2 consisted of 16 severe COVID-19 patients who were admitted to the intensive care unit (ICU; **Table 2**). The aim of this study is to create and validate a neural network that predicts thrombosis risk in COVID-19 patients, using the generated data displayed in **Tables 1**, **2**.

**Table 1:** General characteristics and laboratory tests of the hospital admissions patient (n=133) cohort 1.

		Reference range	COVID-19 patients without thrombosis (n=114)	COVID-19 patients with thrombosis (n=19)	p-value
**General characteristics**	Sex (% male)		64.0%	73.7%	0.028
	Age (years)		64.1 ( ± 14.1)	61.1 ( ± 8.1)	ns
	Mortality (%)		15.8%	10.5%	<0.001
**Inflammation and infection**	anti-SARS-Cov-2 IgM (COI)	0.00-1.00	0.56 ( ± 1.24)	0.51 ( ± 0.59)	ns
	anti-SARS-Cov-2 IgG (COI)	0.00-1.00	8.72 ( ± 12.15)	9.95 ( ± 14.4)	ns
	C-reactive protein (mg/mL)	0 - 5	106 ( ± 84)	182 ( ± 98)	0.001
**Hemostatic parameters and coagulation factors**	APTT (sec)	25 - 33	31.1 ( ± 6.1)	29.1 ( ± 5.1)	ns
	D-dimer (µg/mL)	0.01 - 0.51	1.61 ( ± 2.11)	3.41 ( ± 5.01)	0.046
	Fibrinogen (g/L)	1.81 - 4.51	5.31 ( ± 1.71)	6.11 ( ± 1.71)	0.021
	Protein C (%)	65 - 135	85.1 ( ± 24.1)	83.1 ( ± 21.1)	ns
	Antithrombin (%)	98 - 137	99.1 ( ± 17.1)	105.1 ( ± 11.1)	ns
	α2-macroglobulin (µM)	1.71 - 4.71	4.51 ( ± 1.91)	3.71 ( ± 1.31)	0.032
	VWF (%)	50 - 200	186 ( ± 45)	208 ( ± 36)	0.026
	active VWF (%)	92 - 155	157 ( ± 77)	162 ( ± 42)	ns
	VWF propeptide (%)	73 - 189	216 ( ± 110)	249 ( ± 107)	ns
	FVIII (%)	76 - 237	162 ( ± 79)	208 ( ± 104)	0.005
**Thrombin generation**	ETP (nM·min)	899 - 1697	1238 ( ± 397)	1329 ( ± 550)	ns
	Peak (nM)	185 - 462	211 ( ± 88)	232 ( ± 102)	ns
	Lag time (min)	1.71 - 3.81	4.41 ( ± 1.81)	4.71 ( ± 2.51)	ns
	Time-to-peak (min)	3.21 - 6.61	7.81 ( ± 3.01)	8.41 ( ± 5.41)	ns
	Velocity index (nM/min)	55 - 289	77.1 ( ± 52.1)	89.1 ( ± 56.1)	ns
	Time-to-tail (min)	14.8 – 30.9	24.1 ( ± 7.1)	23.1 ( ± 8.1)	ns
	Curve width (min)	12.8 – 27.7	21.1 ( ± 6.1)	20.1 ( ± 6.1)	ns
	Decay index (nM/min)	39 - 124	54.1 ( ± 30.1)	58.1 ( ± 26.1)	ns
**Thrombin dynamics**	PCtot (nM)	746 - 1335	727 ( ± 253)	750 ( ± 291)	ns
	PCmax (nM/min)	153 - 474	200 ( ± 116)	219 ( ± 119)	ns
	T-AT (nM)	729 - 1279	662 ( ± 240)	690 ( ± 258)	ns
	T-α2M (nM)	16 - 63	42.1 ( ± 26.1)	35.1 ( ± 27.1)	ns
	Thrombin decay capacity (min-1)	0.631 - 1.001	0.601 ( ± 0.111)	0.591 ( ± 0.081)	ns
**Plasmin generation**	EPP (nM·min)	237 - 535	751 ( ± 384)	907 ( ± 583)	ns
	Plasmin Peak (nM)	82 - 132	124 ( ± 30)	123 ( ± 28)	ns
	Plasmin Lag time (min)	3.31 - 8.01	5.21 ( ± 1.71)	5.31 ( ± 1.61)	ns
	Plasmin Time-to-peak (min)	5.01 - 9.71	7.61 ( ± 1.91)	8.11 ( ± 2.41)	ns

Results are shown as the mean ± SD.

**Table 2:** General characteristics and laboratory tests of the intensive care unit patient (n=16) cohort 2.

		Reference range	COVID-19 patients without thrombosis (n=9)	COVID-19 patients with thrombosis (n=7)	p-value
**General characteristics**	Sex (% male)		66.7%	57.1%	ns
	Age (years)		76.1 ( ± 6.1)	56.1 ( ± 13.1)	ns
	Mortality (%)		55.6%	0.0%	0.021
**Inflammation and infection**	anti-SARS-Cov-2 IgM (COI)	0.00-1.00	3.21 ( ± 5.11)	2.81 ( ± 2.51)	ns
	anti-SARS-Cov-2 IgG (COI)	0.00-1.00	31.1 ( ± 9.1)	31.1 ( ± 6.1)	ns
	C-reactive protein (mg/mL)	0 - 5	149 ( ± 89)	215 ( ± 130)	ns
**Hemostatic parameters and coagulation factors**	APTT (sec)	25 - 33	42.1 ( ± 11.1)	42.1 ( ± 10.1)	ns
	D-dimer (µg/mL)	0.01 - 0.51	7.11 ( ± 8.51)	6.31 ( ± 8.71)	ns
	Fibrinogen (g/L)	1.81 - 4.51	5.61 ( ± 1.71)	4.61 ( ± 1.91)	ns
	Protein C (%)	65 - 135	120 ( ± 48)	114 ( ± 47)	ns
	Antithrombin (%)	98 - 137	111 ( ± 38)	104 ( ± 29)	ns
	α2-macroglobulin (µM)	1.71 - 4.71	3.91 ( ± 1.11)	4.21 ( ± 3.31)	ns
	VWF (%)	50 - 200	254 ( ± 26)	229 ( ± 40)	ns
	active VWF (%)	92 - 155	245 ( ± 176)	187 ( ± 86)	ns
	VWF propeptide (%)	73 - 189	316 ( ± 124)	297 ( ± 187)	ns
	FVIII (%)	76 - 237	342 ( ± 81)	317 ( ± 112)	ns
**Thrombin generation**	ETP (nM·min)	899 - 1697	1376 ( ± 261)	1340 ( ± 641)	ns
	Peak (nM)	185 - 462	214 ( ± 80)	159 ( ± 81)	ns
	Lag time (min)	1.71 - 3.81	6.21 ( ± 1.51)	8.01 ( ± 2.71)	ns
	Time-to-peak (min)	3.21 - 6.61	10.01 ( ± 2.01)	12.41 ( ± 3.81)	ns
	Velocity index (nM/min)	55 - 289	67.1 ( ± 43.1)	43.1 ( ± 28.1)	ns
	Time-to-tail (min)	14.8-30.9	27.1 ( ± 8.1)	34.1 ( ± 6.1)	0.050
	Curve width (min)	12.8 - 27.7	21.1 ( ± 8.1)	26.1 ( ± 6.1)	ns
	Decay index (nM/min)	39 - 124	51.1 ( ± 30.1)	29.1 ( ± 15.1)	ns
**Thrombin dynamics**	PCtot (nM)	746 - 1335	849 ( ± 217)	772 ( ± 394)	ns
	PCmax (nM/min)	153 - 474	192 ( ± 99)	137 ( ± 71)	ns
	T-AT (nM)	729 - 1279	753 ( ± 226)	591 ( ± 338)	ns
	T-α2M (nM)	16 - 63	36.1 ( ± 13.1)	29.1 ( ± 22.1)	ns
	Thrombin decay capacity (min-1)	0.631 - 1.001	0.651 ( ± 0.221)	0.661 ( ± 0.191)	ns
**Plasmin generation**	EPP (nM·min)	237 - 535	926 ( ± 441)	1036 ( ± 885)	ns
	Plasmin Peak (nM)	82 - 132	102 ( ± 20)	103 ( ± 60)	ns
	Plasmin Lag time (min)	3.31 - 8.01	6.71 ( ± 1.11)	9.21 ( ± 2.11)	0.011
	Plasmin Time-to-peak (min)	5.01 - 9.71	9.01 ( ± 1.41)	14.51 ( ± 4.51)	0.002

Results are shown as the mean ± SD.

In cohort 1 (**Table 1**), COVID-19 patients with thrombosis were significantly younger than patients without thrombosis, and remarkably, mortality was significantly lower in the thrombosis group. The activated partial thromboplastin time (aPTT) was comparable between the groups, but D-dimer, fibrinogen and CRP were higher in the thrombosis group. α_2_M was significantly lower in the thrombosis group, and FVIII and VWF were significantly higher. Even though thrombin generation, thrombin dynamics and plasmin generation showed trends towards differences between thrombotic and non-thrombotic patients, these differences were not statistically significant. In the ICU cohort 2 (**Table 2**), COVID-19 patients with or without thrombosis did not differ significantly in age, but thrombotic COVID-19 patients were significantly less likely to die during their hospital stay. aPTT, D-dimer, fibrinogen, CRP and other coagulation factors were comparable between the groups. Although thrombin generation, thrombin dynamics and plasmin generation showed trends towards differences between thrombotic and non-thrombotic patients, these differences were not statistically significant, except for the PG lag time.

**Figure 1:**
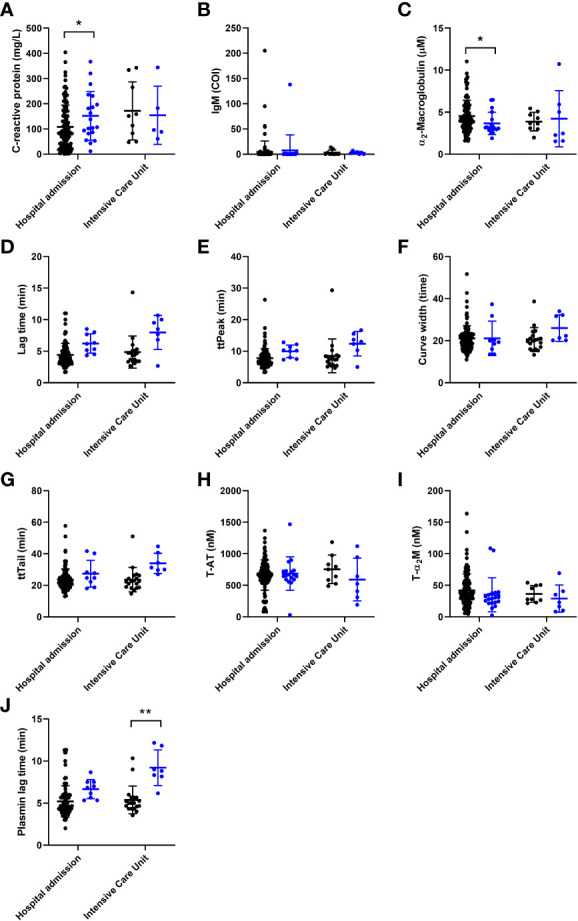
The individual association of coagulation and general (laboratory) parameters used for the development on a neural net for the prediction of thrombosis in COVID-19 patients, arranged from most to least important. Patients without thrombosis are depicted as black circles and patients with thrombosis are depicted as blue circles. **(A)** C-reactive protein was significantly higher in the thrombosis group of the hospital admission cohort. **(B)** Anti-SARS-CoV-2 IgM antibody titer did not differ between the groups. **(C)** α2-macroglobulin was significantly lower in the thrombosis group of the hospital admission cohort. **(D-F)** The width of the TG curve, lag time and time-to-peak did not significantly different between the groups. **(G-I)** The time-to-tail, thrombin-antithrombin formation and thrombin-α2-macroglobulin formation did not differ between the groups. **(J)** The plasmin generation lag time was significantly longer in the thrombosis group of the ICU cohort. The data are represented as mean ± standard deviation. * and ** respectively indicate a p-value smaller than 0.05 and 0.01.

For the neural networking approach, we tested the parameters listed in **Tables 1**, **2** and selected the most promising predictive parameters to achieve the highest predictive accuracy for COVID-19-related thrombosis. **Figure 1** shows in detail the differences of the 10 selected parameters in thrombotic and non-thrombotic COVID-19 patients that contributed significantly to the performance of the neural network. Single thrombin generation parameters could not make a conclusive distinction between thrombosis and non-thrombosis patients, even though a trend is visible toward higher time-dependent variable values in both thrombosis groups, which is borderline significant (**Figures 1B, D, H, I**). ETP and peak height did not differ between thrombotic and non-thrombotic COVID-19 patients (**Table 1**). Thrombin dynamics analysis revealed that thrombin-inhibitor complex formation did not differ significantly between the thrombosis and non-thrombosis groups (**Figures 1E, J**), and comparable amounts of thrombin were formed.

Another important process to maintain the hemostatic balance is the dissolvement of blood clots. A key process in clot lysis is generation of the fibrin cleaving enzyme plasmin. The time until the generation of the first traces of plasmin is significantly longer in thrombosis patients than in COVID-19 patients without thrombosis in the ICU (**Figure 1F**). In contrast to the significant reduction of α_2_M levels in thrombosis patients (**Figure 1C**), inflammatory marker CRP is elevated in COVID-19 patients (**Figure 1A**). The last input that was selected for the neural network was the IgM titer of COVID-19 antibodies because a trend was visible towards a lower IgM titer in thrombotic COVID-19 patients (**Figure 1G**).

**Figure 2:**
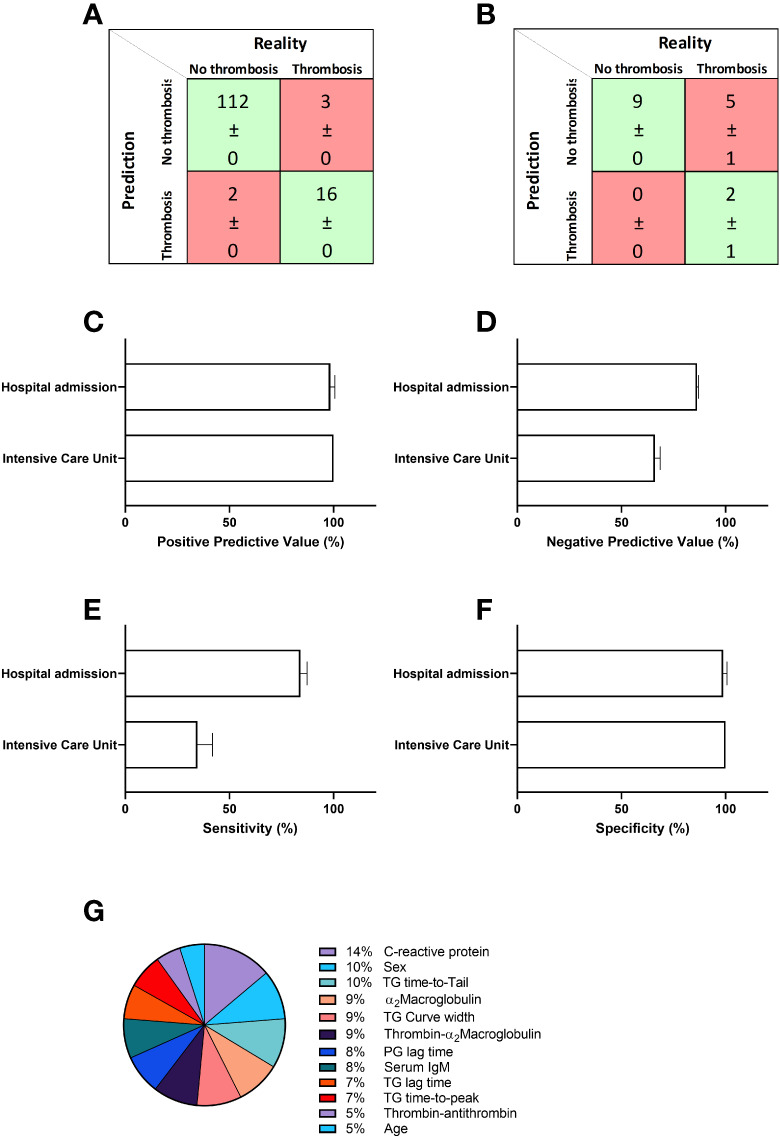
Prediction of thrombosis in hospitalized COVID-19 patients. A neural network was constructed to predict thrombosis based on the input parameters described in table 2 in the first patient cohort. **(A)** The confusion matrix shows that on average 112 out of 114 non-thrombosis patients were correctly predicted and 16 out of 19 were correctly predicted to suffer from thrombosis due to COVID-19. **(B)** Further validation of the network in a second and separate cohort of COVID-19 patients shows the accurate prediction of 9 out of 9 non-thrombosis patients and 2 out of 7 thrombosis patients. **(C)** The positive predictive value (PPV) was 98% for the hospital admission cohort and 100% for the ICU cohort. **(D)** The negative predictive value (NPV) was 86% for the hospital admission cohort and 66% for the ICU cohort. **(E)** The sensitivity was 84% for the hospital admission cohort, and 34% for the ICU cohort. **(F)** The specificity was 99% and 100%, respectively for the hospital admission and ICU cohorts. **(G)** The relative importance for each input variable for the accuracy of the outcome of the neural network. Data are represented as mean ± standard deviation of 10 neural net development runs in the confusion matrices in panel A and B, and in the bar charts in **(C, D)**.

Although analysis of coagulation functionality and immunology did reveal significant differences between thrombotic and non-thrombotic COVID-19 patients, these differences were not striking enough to be used for risk stratification. To integrate the selected data and in order to develop a prediction model for thrombosis, we applied the artificial intelligence method neural networks. We constructed a neural net that predicts COVID-19 related thrombosis based on laboratory parameters collected at the time of hospital admission. **Table 3** summarizes the input variable used for the development of the neural network. The input parameters can be divided into 5 main groups: general characteristics, immunology, thrombin generation, thrombin inhibitors and plasmin generation. General characteristics included age and sex, and general laboratory parameters included IgM titer and CRP levels. Coagulation variables were subdivided in three groups: Thrombin generation parameters (lag time, ttPeak, ttTail, and curve width), thrombin inhibitors variables (α_2_M, T-α_2_M and T-AT) and plasmin generation. A neural network with 10 hidden neurons was trained in a randomly selected subset (70%) of the samples of the hospital admission cohort. The remaining 30% of samples were randomly divided between the validation (15%) and testing (15%) dataset. Network development was performed in 10-fold and the average results ( ± SD) are shown in **Figure 2**. In the non-thrombosis group, on average 112 out of 114 patients were correctly predicted to stay thrombosis-free, whereas in the thrombosis group, 16 out of 19 patients were correctly predicted to suffer from thrombosis during their hospital stay (**Figure 2A**). In the hospital admission cohort, the positive predictive value (PPV) was 98% (**Figure 2C**) and the negative predictive value (NPV) was 86%. The overall accuracy of the neural network was 91%.

**Table 3:** Input parameters for the neural network.

Category	Specific parameters
**General characteristics**	Age
	Sex
**Immunology**	C-reactive protein
	IgM titer for COVID-19
**Thrombin generation**	Lag time
	Time-to-peak
	Time-to-tail
	Curve width
**Thrombin inhibitors**	α_2_-Macroglobulin
	T-α_2_-Macroglobulin complexes
	T-antithrombin
**Plasmin generation**	Plasmin lag time

We tested the model further in a second, separately acquired COVID-19 patient cohort. In this cohort, 9 out of 9 non-thrombotic COVID-19 patients were correctly predicted and 2 out of 7 thrombotic patients were classified correctly as patients that would suffer from a thrombosis during their hospital stay (**Figure 2B**). In the ICU cohort, the positive predictive value was 100% (**Figure 2C**) and the negative predictive value (NPV) was 66%. In **Figure 2E** we quantified the relative importance of each input parameter to the predictive accuracy of the neural network. CRP levels contribute the most to the predictive accuracy (14%). Both sex and ttTail contribute equally by 10%, followed by 9% contribution each of α_2_M, curve width and T-α_2_M. Plasmin generation lag time and serum IgM both contribute 8% and TG lag time and ttPeak contribute 7%. The last 10% is divided between T-AT (5%) and age (5%).

## Discussion

COVID-19 patients show very diverse pathogenesis varying from mild flu-like symptoms to hospitalization, ICU admission or even death (4, 24). An important complication in COVID-19 pathogenesis is the development of thrombosis (25, 26). Many research has been conducted to study the disease process aiming to predict its severity (27–29). As COVID-19 can have a very divergent course of disease, it is of interest to predict the risk of COVID-19 severity and the risk of complications on an individual level. In this study we set out to develop a prediction algorithm for thrombosis in COVID-19 patients using the machine learning method neural networks.

We enrolled two populations of confirmed COVID-19 patients. Thrombotic complications were registered in detail for both cohorts (19, 20). Our study revealed that COVID-19 patients that suffer from COVID-19 related thrombosis have a trend towards higher TG, reduced levels of thrombin inhibitor α_2_M, and an elevated plasmin generation lag time. Although previous reports have shown reduced AT levels in severe COVID-19 patients, we did not observe a difference in AT in patients with or without thrombosis, although there seemed to be a trend toward lower AT levels in patients with thrombosis (1). The observed changes in hemostasis point towards a more prothrombotic phenotype, as more thrombin is formed, thrombin is inhibited less efficiently, and it takes longer until the clot is dissolved. Additionally, we found that CRP was increased, as reported by others (30). Interestingly, the mortality rate in subjects with COVID-19 related thrombosis is lower than the mortality rate in patients without thrombosis. This was proposed to be attributable to differences in age and treatment between the groups (20, 31) as older individuals are known to be at risk for severe COVID-19 complications (32), including thrombosis (33).

Even though we found differences in biomarkers of hemostasis between thrombotic and unaffected patients, the differences in test results were not pronounced enough to use a single biomarkers as an independent predictor of thrombosis in COVID-19 patients. Therefore, we used machine learning to integrate all variables into a neural net that accurately classifies COVID-19 patients with a high risk of future thrombosis at the time of hospital admission. The accuracy of the neural net is very high (91%). Especially the high positive predictive value of 98% and specificity of 99% would be very useful in the risk stratification of patients in order to treat patients with a high thrombosis risk or to avoid profound anticoagulation in COVID-19 patients at low risk for thrombosis. The neural net showed a higher PPV and specificity (both 100%) in cohort 2 that was used for the external validation of our model, indicating that the neural net can also be used for risk stratification in other COVID-19 patient cohorts.

Although many neural networks have been constructed for COVID-19 patients to predict individual risks (8–13) or even forecast the spread of the virus (14–17), this is the first network to our knowledge to accurately identify patients at high risk for COVID-19 related thrombosis. The most important contributors to the neural network to predict thrombosis are CRP, sex, thrombin inhibition by α_2_M (α_2_M levels and T-α_2_M formation), and the time that thrombin is present in clotting plasma (curve width and time-to-tail). The time until the first traces of thrombin and plasmin are formed is important for the accuracy of the model and thrombin-antithrombin complex formation and age are of minor importance. CRP is known to be related to disease severity (34), and sex has been reported to be an important influencer of the course of the disease since the start of the pandemic (35). Another important factor, plasma α_2_M levels, is both an acute phase reactant and a thrombin inhibitor (36), and to our knowledge has not previously been investigated in COVID-19 patients. Interestingly, age is of less importance than expected to the neural net, even though others have shown that the elderly have an increased risk for severe COVID-19 and COVID-19 related thrombosis. This could potentially be a result of the high overall age of both cohorts, although the cohorts represent a cross section of the population of hospitalized patients.

Additionally, this studies has two limitations, being (1) the limited sample size of the cohorts for the neural networking approach, and (2) that several input parameters for the neural net are not readily available for routine clinical laboratories. However, the developed neural net was not accurate if the thrombin generation and thrombin dynamics parameters were excluded, as the thrombin generation test is a global hemostasis test, and therefore an important predictor of thrombotic risk (37). Moreover, recent advances in the thrombin generation method have led to the development of a fully automated device to measure thrombin generation in the clinical setting (38).

In conclusion, we developed a neural network that predicts future thrombosis in COVID-19 patients at the time of hospital admission with a positive predictive value of respectively 98% and 100% in a general hospital admission and ICU cohort. A combination of general (laboratory) parameters and hemostatic markers can predict future COVID-related thrombosis, whereas the separate variables showed no or slight differences between thrombotic and non-thrombotic patients.

## Data availability statement

The original contributions presented in the study are included in the article/supplementary material. Further inquiries can be directed to the corresponding author.

## Ethics statement

The studies involving human participants were reviewed and approved by MEC-U” and the “Comité Medische Ethiek” medical ethics committees. The patients/participants provided their written informed consent to participate in this study.

## Author contributions

RL-K and BL conceived and designed the study and co-wrote the manuscript. RL-K performed experiments, analyzed the data and performed the neural network analyses. MN performed experimental work and analysis. RJ, RF, AF, and JR performed and supervised the patients sample collection. All authors contributed to the article and approved the submitted version.

## Conflict of interest

RL-K, MN, and BL declare no competing interests and are employees of Synapse Research Institute, part of Diagnostica Stago S.A.S.

The remaining authors declare that the research was conducted in the absence of any commercial or financial relationships that could be construed as a potential conflict of interest.

## Publisher’s note

All claims expressed in this article are solely those of the authors and do not necessarily represent those of their affiliated organizations, or those of the publisher, the editors and the reviewers. Any product that may be evaluated in this article, or claim that may be made by its manufacturer, is not guaranteed or endorsed by the publisher.
